# Complete Genome Sequences of Eight Phages Infecting Enterotoxigenic Escherichia coli in Swine

**DOI:** 10.1128/MRA.00858-20

**Published:** 2020-09-03

**Authors:** Alice Ferreira, Hugo Oliveira, Daniela Silva, Carina Almeida, Julia Burgan, Joana Azeredo, Ana Oliveira

**Affiliations:** a CEB (Centre of Biological Engineering) LIBRO (Lab. de Investigação em Biofilmes Rosário Oliveira), University of Minho, Braga, Portugal; b ALS Controlvet, Tondela, Portugal; DOE Joint Genome Institute

## Abstract

We report eight phages infecting enterotoxigenic Escherichia coli responsible for intestinal infections in piglets. Phages vB_EcoM_F1, vB_EcoM_FB, vB_EcoS_FP, vB_EcoM_FT, vB_EcoM_SP1, vB_EcoP_SP5M, vB_EcoP_SP7, and vB_EcoS_SP8 were isolated between 2007 and 2018 in the Iberian Peninsula. These viruses span the three tailed phage families, *Podoviridae*, *Siphoviridae*, and *Myoviridae.*

## ANNOUNCEMENT

Enterotoxigenic Escherichia coli (ETEC) infections cause diarrhea and death among weaning and postweaning piglets (1). Resulting economic losses have encouraged the swine industry to find alternatives to prevent and treat such infections (2). Bacteriophages have been proposed as an effective solution (3).

Here, we present eight complete E. coli phage genome sequences isolated from samples collected from poultry and pig farms between 2015 and 2018 by ALS-Controlvet across Portugal and Spain (Table 1). The exception was vB_EcoM_F1, previously phiF38E (4), isolated in 2007. Samples (feces, poultry litter, and sewage) were stored at 4°C until processing. For phage amplification, samples and E. coli field isolates (Table 1) were incubated overnight in LB broth at 37°C, and filtrate was plated over 0.6% (wt/vol) LB agar. Phage isolation and production followed standard propagation and amplification techniques (4). DNA was extracted from a highly concentrated phage suspension using the phenol-chloroform method, as presented before (5). Phage DNA libraries were prepared with a KAPA HyperPlus or Nextera XT library preparation kit and sequenced in an Illumina MiSeq platform (300-bp paired-end sequencing reads). Data quality was controlled with FastQC v0.11.5 (6). Reads were trimmed with BBDuk and *de novo* assembled with the Geneious assembler (medium-low sensitivity option) in Geneious Prime v2020.1 software (7). Genomic termini were evaluated in PhageTerm with default parameters, but we could not determine the genome ends (8). The genomes were annotated in RAST MyRAST (9) and complemented or manually verified (default settings) with BLAST (10), tRNAscan-SE v2.0 (11), ARAGORN (12), and HHpred (13) (E value cutoff, 1 × 10^–5^; query coverage, ≥80%).

**TABLE 1:** E. coli phages, GenBank accession numbers, origin, genome characteristics, and closest published phages

Phage designation	Host strain	Morphology	Isolation source	Location	DNA library prepn kit	Coverage (×)	Genome length (bp)	No. of coding sequences	G+C content (%)	Closest phage deposited in GenBank	SRA accession no.	GenBank accession no.
vB_EcoM_F1	Host phage 1	*Myoviridae*	Poultry sewage	Portugal	KAPA HyperPlus	125	168,410	279	35.4	YUEEL01	SRR12228473	MT682712
vB_EcoM_FB	Host phage B	*Myoviridae*	Poultry beds	Spain	KAPA HyperPlus	134	171,555	271	39.4	QL01	SRR12228472	MT682711
vB_EcoS_FP	Host phage P	*Siphoviridae*	Poultry beds	Spain	KAPA HyperPlus	131	43,757	68	44.1	vB_EcoS-2862V	SRR12228471	MT682706
vB_EcoM_FT	Host phage T	*Myoviridae*	Poultry beds	Spain	KAPA HyperPlus	134	167,431	267	35.3	SHFML-11	SRR12228470	MT682710
vB_EcoM_SP1	SP16	*Myoviridae*	Sewage water	Portugal	Nextera XT	292	165,416	273	35.6	vB_EcoM_IME340	SRR12228469	MT682709
vB_EcoP_SP5M	SP36	*Podoviridae*	Sewage water	Portugal	Nextera XT	765	72,896	88	43.2	vB_EcoP_PhAPEC7	SRR12228468	MT682708
vB_EcoP_SP7	SP36	*Podoviridae*	Sewage water	Portugal	Nextera XT	301	39,305	53	50.1	ST31	SRR12228467	MT682707
vB_EcoS_SP8	SP22	*Siphoviridae*	Sewage water	Portugal	Nextera XT	938	57,585	84	43.3	SE1	SRR12228466	MT682705

**FIG 1:**
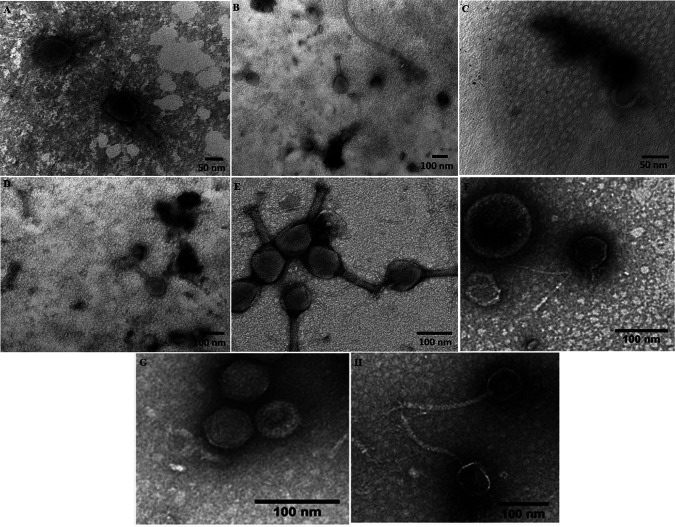
Transmission electron micrographs of E. coli phages. Microscopy showing the virion particle morphology (stained with 2% uranyl acetate) of phages vB_EcoM_F1 (A), vB_EcoM_FB (B), vB_EcoS_FP (C), vB_EcoM_FT (D), vB_EcoM_SP1 (E), vB_EcoP_SP5M (F), vB_EcoP_SP7 (G), and vB_EcoS_SP8 (H). The scale bar represents 50 nm in images A and C and 100 nm in images B, D, E, F, G, and H.

All eight phages are tailed and have double-stranded DNA genomes. Four of them are from *Myoviridae*, two are from *Siphoviridae*, and two are from the *Podoviridae* family (Fig. 1). The different genome lengths (39,305 bp to 171,155 bp) with 53 to 279 coding sequences, 0 to 10 tRNAs, and G+C contents from 35.1% to 50.1% reflect the diversity of the genomes obtained.

The eight genomes’ alignment (NCBI tool BLASTn) revealed high homology with several viruses from the nonredundant database (Table 1). Phages vB_EcoM_F1, vB_EcoM_FB, vB_EcoM_FT, vB_EcoM_SP1, and vB_EcoS_SP8 showed 95% overall nucleotide identity and genome coverage of approximately 90%, while vB_EcoS_FP, vB_EcoP_SP5M, and vB_EcoP_SP7 showed >95% homology and a coverage of 80%. All phages encoded >50% unknown proteins, a large terminase subunit, and a DNA helicase protein. Also, the majority identified a putative small terminase subunit, a DNA primase, a capsid vertex protein, a DNA polymerase, and a portal protein. The genome analysis also enabled the identification of lytic proteins—endolysin and holin. Six phages’ endolysins harbor a predicted *N*-acetylmuramidase domain, while vB_EcoP_SP7 possesses a putative *N*-acetylmuramoyl-l-alanine amidase. Here, a spanin complex was also identified, comprising inner and outer membrane subunits. Interestingly, the vB_EcoS_SP8 genome did not identify any lysis-related protein.

The analyses of these phages’ genomes together with additional studies focused on their fitness can provide new resources to combat ETEC infections.

### Data availability.

The GenBank accession numbers of the E. coli phage genome sequences are listed in Table 1. The Sequence Read Archive data for the genomes are available under BioProject accession number PRJNA646048.
